# Supplementary material for: Guanine quadruplexes mediate mitochondrial RNA polymerase pausing
Source: bioRxiv. 2023 Oct 19:2023.10.17.562821. Preprint. [Version 1] doi: 10.1101/2023.10.17.562821 (PMC10614896; doi:10.1101/2023.10.17.562821)
Supplement: Supplement 1 [file media-1.pdf]

Guanine quadruplexes mediate mitochondrial RNA polymerase pausing

Ryan Snyder[1], Don Delker[2], Joshua T Burdick[3], Vivian G. Cheung[3,4], Jason A. Watts[1,5]

[1]Epigenetics and Stem Cell Biology Laboratory, National Institute of Environmental Health Sciences, National Institutes of Health, Research Triangle Park, NC 27709, USA
[2]Integrative Bioinformatics, National Institute of Environmental Health Sciences, National Institutes of Health, Research Triangle Park, NC 27709, USA
[3]Life Sciences Institute, University of Michigan, Ann Arbor, MI 48109, USA
[4]Department of Pediatrics, Division of Neurology, University of Michigan, Ann Arbor, MI,USA.
[5]Department of Medicine, University of Michigan, Ann Arbor, MI 48109, USA;

Supplemental Figures 1-4
Supplemental Table 1

Supplemental Figure 1

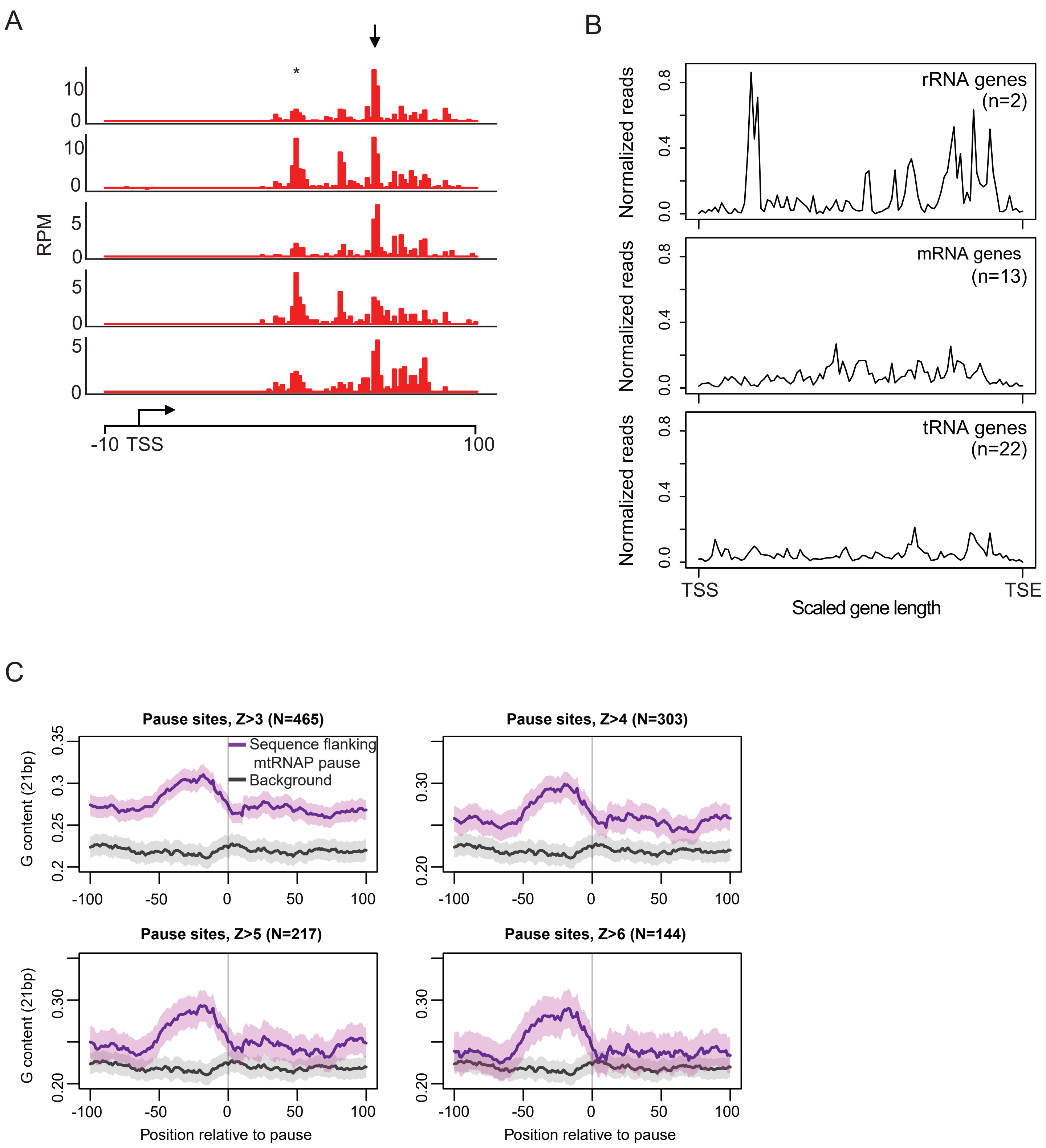

Supplemental Figure 1. mtRNAP pauses proximal to the light strand promoter (A) PRO-seq coverage on the light strand in primary fibroblasts (N=5). The TSS (arrow) and known site of mtRNAP pausing (*) are indicated. (B) Metagene plots of the mtRNAP distribution at rRNA, mRNA, and tRNA genes indicate mtRNAP pausing is not enrichment near the beginning or end of transcripts. (C) Guanine content near pause sites at increasingly stringent Z-score threshold to call pause sites, as compared to background sequence where mtRNAP does not pause (black).

Supplemental Figure 2

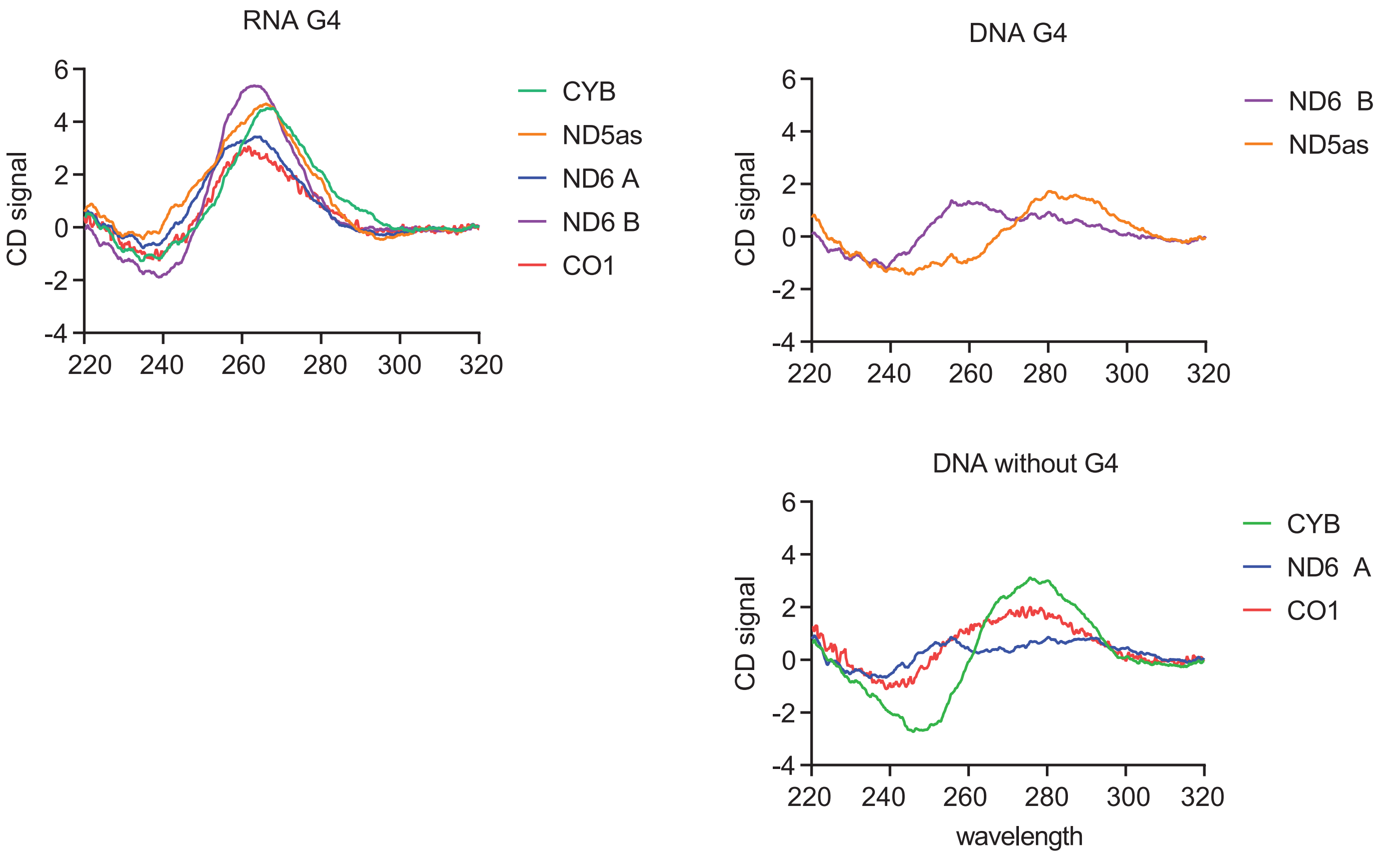

Supplemental Figure 2. G4 formation is more stable in RNA. Circular dichroism spectra of RNA or DNA oligos. Spectra from RNA oligos are consistent with parallel quadruplexes. DNA oligos are heterogenous with parallel or antiparallel quadruplexes (upper plot) or unfolded molecules (lower plot).

Supplemental Figure 3

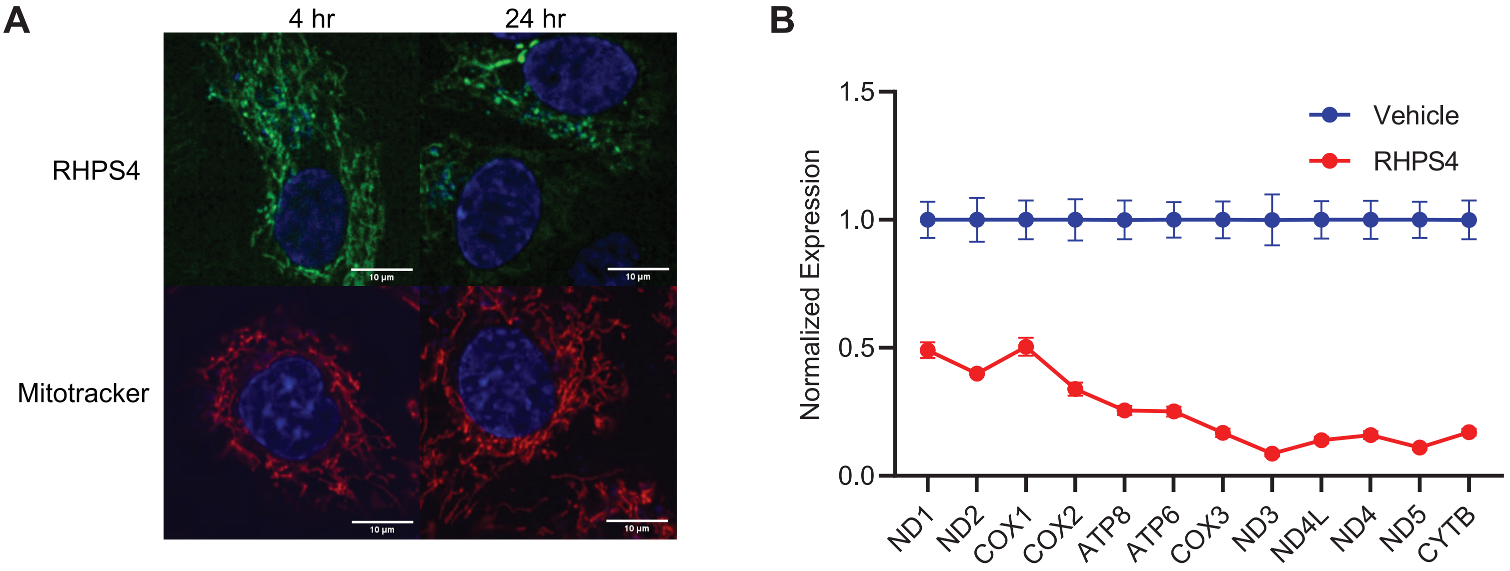

Supplemental Figure 3. mtRNAP transcription is decreased when G4 are stabilized. (A) Confocal image showing RHPS4 (green) uptake into mitochondria following 24 hours of drug treatment. (B) Mitochondrial gene expression in RPTEC before and 24 hours after RHPS4 treatment normalized to vehicle treated cells. Gene expression is in arbitrary units (N=3; $P<0.0001$, one-sided ANOVA, error bars=S.E.M.).

Supplemental Figure 4

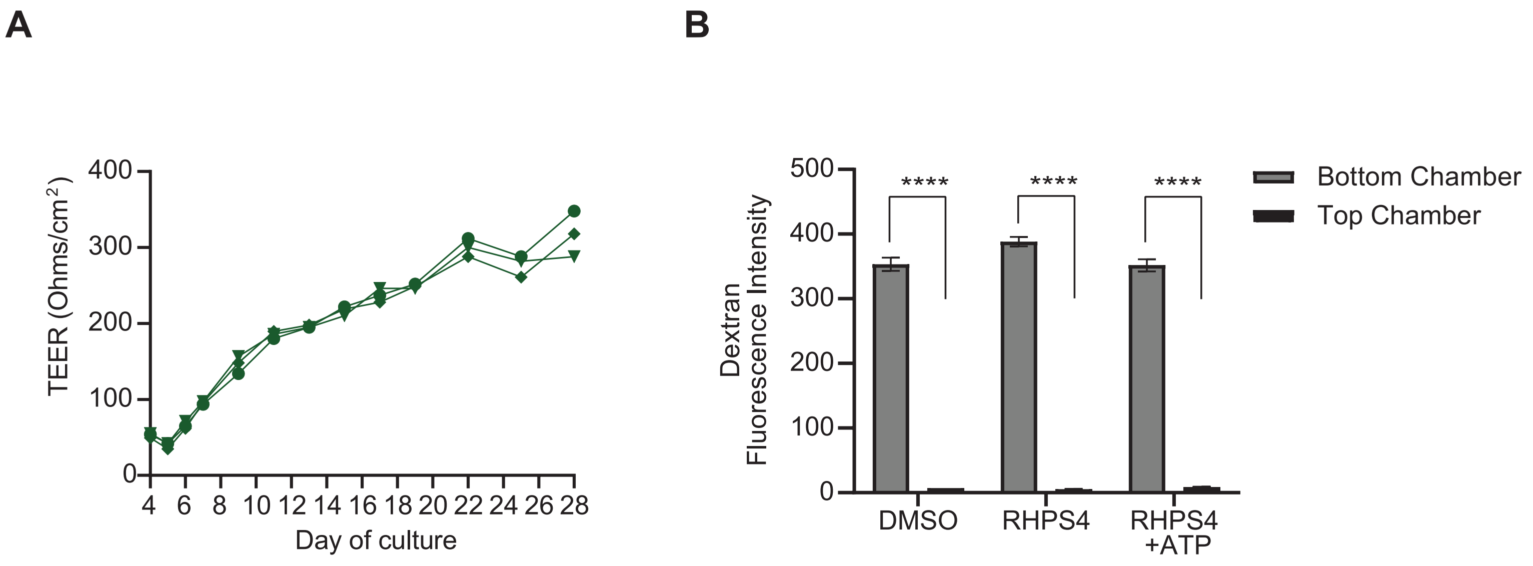

Supplemental Figure 4. RPTEC monolayers form a barrier to diffusion. (A) 5-fold increase in transepithelial electrical resistance (TEER) measurements over 30 days of culture (N=3, P<0.0001; one-way ANOVA). (B) RPTEC monolayers prevent diffusion of fluorescently labelled dextran from the bottom chamber to the top chamber in a transwell.

Supplemental Table 1: Oligonucleotides

Oligo Name:	Sequence:
CO1_ChIP_F	TACCTCCCTCTCTCCTACTCCT
CO1_ChIP_R	GGCGTTTGGTATTGGGTTATGG
CO1_RNA	UGGAGGCCGGAGCAGGAACAGGU
CO1_RNA_mut	UUUAUUCCUUAGCAUUAACAUUU
CO1_DNA	TGGAGGCCGGAGCAGGAACAGGT
CYB_RNA	CGGGCGAGGCCUAUAUUACGGA
CYB_DNA	CGGGCGAGGCCTATATTACGGA
ND5AS_RNA	AGGCCUAGAUAGGGGAUUGUGCGGUGUGUGAUG
ND5AS_DNA	AGGCCTAGATAGGGGATTGTGCGGTGTGTGATG
ND6A_RNA	UGGAGGUAGGAUUGGUGCUGUGGGU
ND6A_DNA	TGGAGGTAGGATTGGTGCTGTGGGT
ND6B_RNA	UGAUGGGGUGGUGGUUGUGGU
ND6B_DNA	TGATGGGGTGGTGGTTGTGGT
